# Facile nucleophilic substitution approach for the spectrofluorimetric assay of natamycin based on diarylpyrrolone formation, evaluation of method greenness

**DOI:** 10.1186/s13065-025-01388-3

**Published:** 2025-01-24

**Authors:** Sayed M. Derayea, Fatma F. Mohammed

**Affiliations:** Analytical Chemistry Department, Faculty of Pharmacy, Minia University, Minia, Egypt

**Keywords:** Natamycin, Nucleophilic substitution reaction, Spectrofluorimetry, Aqueous humour, Fluorescamine

## Abstract

An ecofriendly, effective, and selective spectrofluorimetric approach for natamycin analysis was developed using fluorescamine as a fluorogenic probe. Natamycin is the only topical ocular antifungal medication that is presently on the market for treating keratitis, conjunctivitis, and blepharitis caused by yeast and other fungi. Owing to its primary aliphatic amino group, natamycin can easily interact with fluorescamine resulting in the formation of the highly fluorescent diaryl pyrrolone derivative. The derivatization reaction was completed within very short time at room temperature in borate buffer solution (pH 7.6). The fluorescence intensity of the reaction product was monitored at 465 nm after exciting at 390 nm. The linearity range of the spectrofluorimetric method was 0.25–4.0 µg/mL of natamycin with limit of detection (LOD) of 0.082 µg/mL. The method was applied for the determination of the cited drug in pharmaceutical eye drops and artificial aqueous humor with high percentage recoveries and low relative standard deviations. In addition, the involved analytical procedure was green based on the results of the ecology scale scores.

## Introduction

**Fig. 1:**
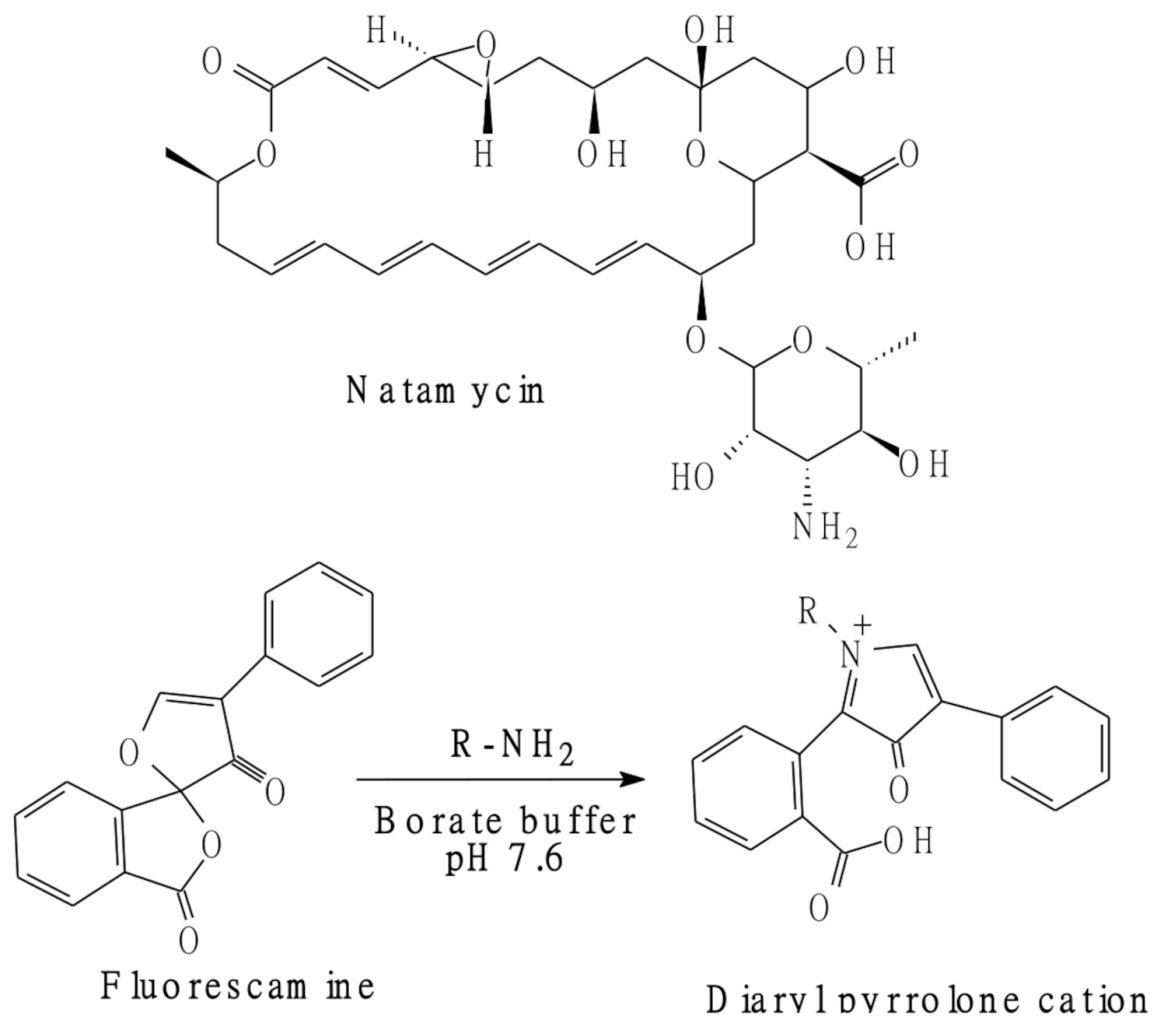
Chemical structure of natamycin and the reaction of fluorescamine with primary amines to yield the fluorescent product (diaryl pyrrolone cation)

The interaction of fluorescamine (Fig. 1) as a fluorogenic reagent can be completed within reasonable timescale and does not need any tedious fine alteration of reaction conditions. The reaction in most cases proceeds within few minutes in neutral or slightly alkaline media. The reagent is selective for primary amines and has versatile application in the assay of protein peptides, amino acids and pharmaceutical compounds.

Natamycin (Fig. 1), is an antifungal drug of polyene group. It can treat fungal infections, in addition to those infections caused by protozoa and algae, but it does not show any activity towards bacteria. Natamycin is used in topical preparations for treating mucous membranes and skin infections. Moreover, the compound is one of the food additives which is commonly utilized as antimycotic agent for meat and dairy products [1]. Its great pharmaceutical importance comes from being the only currently available topical ophthalmic antifungal preparation. Hence, It is applied for the treatment of yeast and fungal blepharitis, conjunctivitis and keratitis [2].

Various analytical methods have been reported for the determination of natamycin. Most of these methods were chromatographic methods including High performance liquid chromatography (HPLC) [3–9], Ultraperformance liquid chromatography (UPLC) [10] and Thin Layer Chromatography (TLC) [11]. In addition, spectrophotometric [12–15], spectrofluorimetric [16–19] atomic absorption [20], electrophoretic [21], and electrochemical [22–25] methods were also reported.

Although chromatographic methods have adequate sensitivity, these methods suffer from some limitations including the use of large volumes of highly pure organic solvents which increase both the cost of the analysis and the risk of environmental pollution. In addition, samples for HPLC and UPLC should undergo tedious and lengthy pretreatment steps. In some cases, extremely expensive mass detector was also used [5, 7, 10]. Meanwhile the spectrophotometric methods had low sensitivity [12–14]. On the other hand, spectrofluorimetric technique is very simple and it is characterized by its high sensitivity and selectivity. However, the recently reported spectrofluorimetric methods either needed multiple reagents preparation [16], or long reaction time and a heating step that opposed the methods’ simplicity [16, 17]. While the method that utilized nitrogen and sulfur carbon nano dots suffered from limited sensitivity [18].

Although fluorescamine has the ability to react with a wide variety of nucleophilic compounds (alcohols, amines and thiols), fluorescamine gives only fluorescent products in the case of primary amine containing compounds. The reagent itself is a non-fluorescent compound but upon its interaction with primary amine it is converted into a highly fluorescent derivative. This property makes the compound an ideal fluorogenic reagent for analyzing free amine group containing compounds, especially in pharmaceuticals, amino acids, proteins and peptides. Hence it was widely utilized for the determination of a variety of analytes that contain amino groups [26].

The present study aimed to set up a new, fast and simple spectrofluorimetric protocol for natamycin assay based on interacting its primary amine with fluorescamine in buffered medium. The method was utilized for the determination of the cited drug in pharmaceutical eye drops and artificial aqueous humor.

## Experimental

### Instruments

Perkin Elmer LS 45 Luminescence spectrometer (UK) instrument was used for scanning the fluorescence spectra. The spectrometer was controlled by the FL WINLAB™ software loaded on PC. AD11 waterproof pH meter (Hungary) was also used.

### Chemicals and reagents

Natamycin pure powder was kindly brought from Orchidia Company for Pharmaceutical Industries (Cairo, Egypt). No further treatment was done to the powder before its usage.

Fluorescamine, was purchased from (Alfaaesar, Thermo-Fisher, Germany) and (Sigma–Aldrich Chemie GmbH, Germany). The reagent was dissolved in acetone to give concentration of 200 µg/mL. The other chemicals, including acetone, acetonitrile, ethanol, dimethylformamide, methanol, albumin, boric acid, hydrochloric acid, sodium lactate, sodium ascorbate, and sodium hydroxide, were purchased from El Nasr Chemical Co, Cairo, Egypt. All these chemicals were of analytical grade. Preparation of borate buffer solutions was carried out using appropriate volumes of 0.1 M sodium hydroxide and 0.1 M boric acid to get the desired pH value.

The studied pharmaceutical dosage form was Hosaptam^®^ eye drops. It was a suspension that was labelled to contain 5% g w/v of natamycin. It was manufactured in Kahira Company for Pharmaceutical Industries, Cairo, Egypt.

### Preparation of standard drug solution

A stock natamycin solution (250 µg/mL) was prepared by dissolving 25 mg of the drug in 100 mL methanol. After that, different volumes were taken from the stock solution and were diluted with methanol to give the required concentrations (2.5–40 µg/mL). The latter solutions were subjected to analysis using the general procedure.

### General analytical procedure

1.0 mL portions of natamycin working standard solutions (2.5–40 µg/mL) were taken into 10 mL volumetric flasks to give final concentrations in the range (0.25–4.0 µg/mL). Then 0.5 mL of borate buffer solution (pH 7.6) was added. After that, 1.6 mL of fluorescamine solution (200 µg/mL) was added. Ethanol was then added to complete the volume to 10 mL. After waiting for 6 min, the fluorescence of the resulting solution was monitored at 465 nm after being excited at 390 nm. A blank solution was performed similarly without adding the drug solution.

### Procedure for dosage form (eye drops) analysis

Initially, the suspension of the eye drop was shaken well, and then 0.1 mL (which was equivalent to 5 mg of natamycin) was taken and sonicated with small volume of methanol for 5 min. The mixture was diluted with methanol in 100 mL volumetric flask and then filtered to give a solution containing 50 µg/mL of natamycin. The obtained filtrate was diluted to give solutions having concentrations in the recommended analytical range. The procedure of the general assay was carried out on portions of the resulting solutions.

### Analysis of the drug in aqueous humour

Artificial aqueous humour solution was prepared according the previously published procedure [31]. In brief, 6.19 g sodium chloride, 0.39 g potassium chloride, 0.28 g calcium chloride, 2.9 g sodium bicarbonate, 0.8 g glucose, 0.1 g urea 0.1 g albumin, 0.28 g sodium lactate and 0.15 g sodium ascorbate were dissolved in 1 L of water. Then the pH was adjusted by 1.0 M HCl to render the pH 7.2. Finally, the solution was filtered and stored at 20º C until analysis. Into 5 mL screw capped tube, 1.0 mL of the artificial aqueous humor was transferred and mixed with with different volumes of natamycin solution (100 µg/mL) to give final drug concentration in the recommended analytical range. The solutions were completed to 5 mL with methanol and subjected to centrifugation at 3000 rpm for 15 min. After that 1.0 mL from the obtained supernatants were transferred to 10 mL volumetric flasks and were analyzed using the general procedure.

### Determination of reaction stoichiometry

The reaction stoichiometry was estimated by applying Job’s method for continuous variations. Both the drug and fluorescamine were prepared in concentration of 1.2 × 10^− 4^ M in acetone and methanol, respectively. Several solutions that contained varying concentrations of both the reagent and the drug were constituted keeping their total concentration constant. After that, 0.5 mL of borate buffer solution was added, and the general procedure was conducted as described previously. Blank experiments were also conducted that contained different concentrations of the reagent without adding the drug solution. The obtained fluorescence intensity after subtracting that of the blank were plotted versus the mole fractions of natamycin to get Job`s plot.

## Results and discussion

The use of simple and rapid reactions is of a great importance in the field of analytical chemistry because the use of such reactions saves effort and expenses. It is also advantageous to aid in the context of striving to preserve the environment and protect natural resources through the development of new ecofriendly methods.

**Fig. 2:**
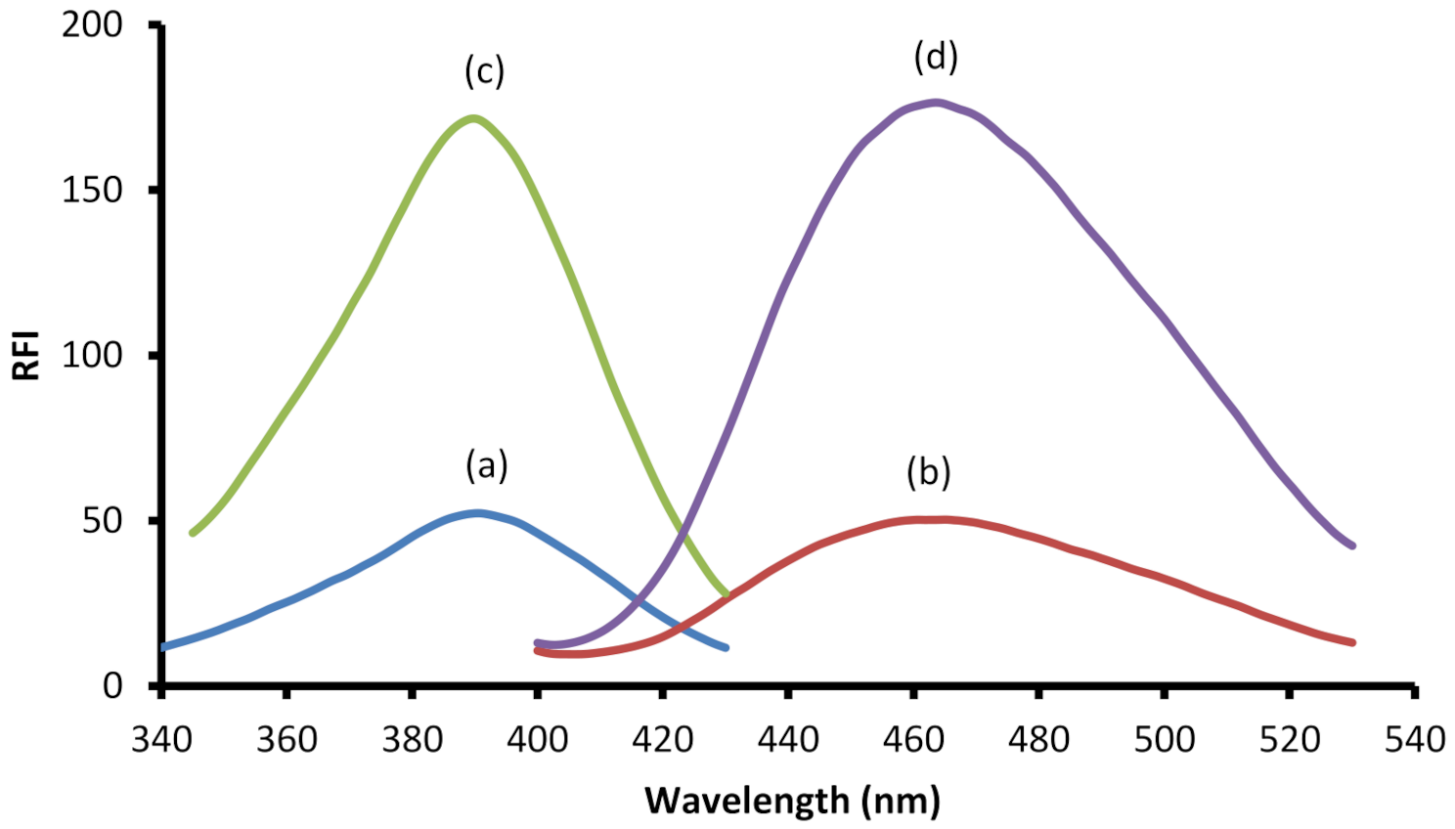
The excitation and emission spectra of both the reagent blank (a & b) and the product of the reaction between the 1.5 µg/mL natamycin and fluorescamine (c & d), showing excitation at 390 nm and emission at 465 nm

The aliphatic and non-planer structure of natamycin make the drug itself doesn’t exhibit native fluorescence and hence there is no direct spectrofluorimetric method for its determination. This necessitated the need to search for a suitable approach that could enable the cited drug analysis using such technique because of its high simplicity and sensitivity. The presence of a primary amino group in the structure of the drug enabled its chemical derivatization using fluorescamine reagent. Fluorescamine is one of the most widely applied fluorogenic reagents for the analysis of primary amine containing compounds. Although there are several fluorogenic reagents for primary amine probing, fluorescamine is the only reagent which itself is not fluorescent and its hydrolytic product is non-fluorescent too. Its reaction is rapid, in a one pot and does not require elevated temperature as the previously reported methods [16, 17]. The procedure does not involve multistep reactions or drastic conditions. In the current work, the reaction of fluorescamine with natamycin was carried out in a buffered medium (pH 7.6) and the formed derivative had a yellow color. As shown in Fig. 2, the formed product exhibited fluorescence activity at 465 (λ _ex_ at 390 nm).

The diaryl-2-hydroxy-pyrrolinone derivative was formerly proposed to be the fluorescent product of fluorescamine interaction with primary amine. However, recent studies [27, 28] proved that the derivative is a pyrrolone cation because its structure is highly conjugated, completely planner and rigid. While the previously claimed hydroxyl pyrrolinone is not planar and less rigid as well as its pyrrolinone ring is not conjugated. The highly conjugated and planar structure is the responsible cause for the fluorescence activity of the pyrrolone derivative. An illustration for the possible reaction pathway of fluorescamine with primary amine is shown in Fig. 1.

### Optimizing the experimental conditions

Optimization of the various experimental conditions was done by changing each variable individually while keeping the other parameters constant. The examined variables included pH, volume of buffer and fluorescamine, time of reaction, and dilution solvents.

#### Influence of buffer pH and volume

**Fig. 3:**
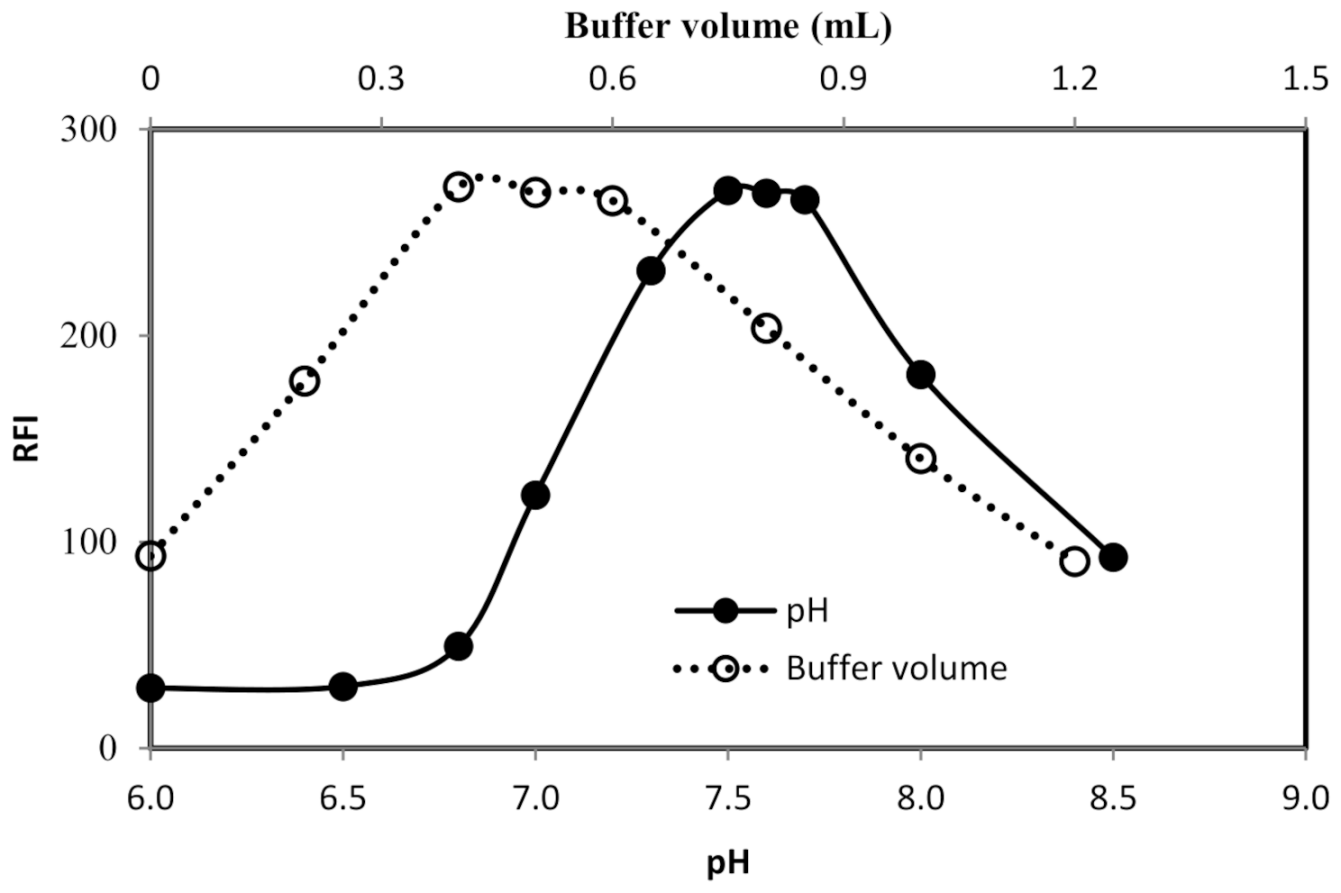
Effect of pH and volume of borate buffer on the RFI of the product of the reaction between natamycin (2 µg/mL) and fluorescamine reagent

**Fig. 4:**
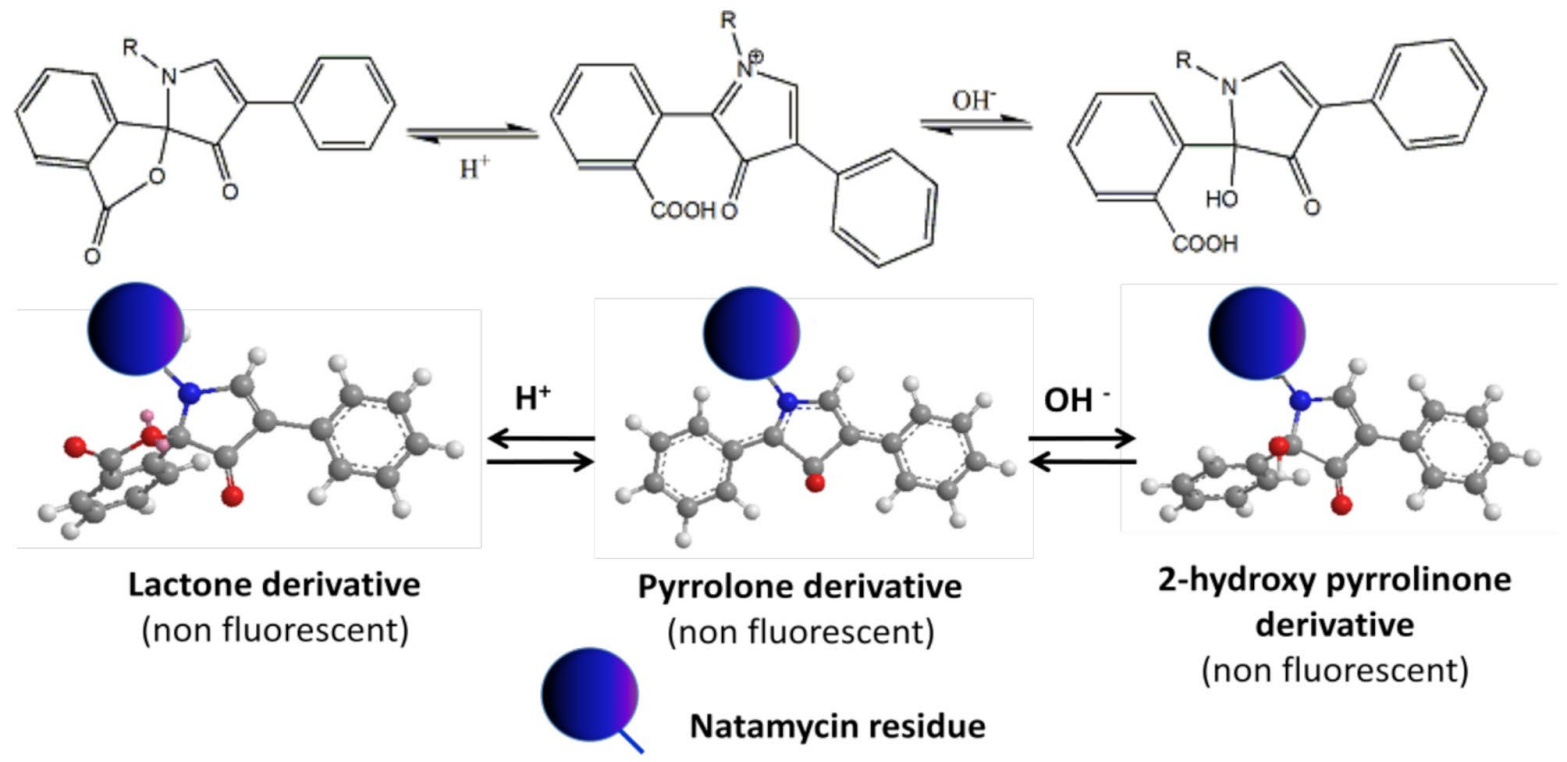
Effect of pH on the chemical structure of the formed product between natamycin and fluorescamine reagent illustrated by 2D and 3D plots

The interaction of fluorescamine with primary amines should be performed in a solution having a specific pH value. Thus, the derivatization procedure was performed in the presence of borate buffer solution having varied pH values (Fig. 3). Maximum fluorescence intensity was obtained when the reaction medium was neutral (pH 7.6 ± 0.1). The fluorescence intensity gradually decreased when the pH moves away from this range (7.5–7.7). The reason for the reduction of the fluorescence outside this pH range may be due to structural changes of the formed product upon changing the pH of the medium (Fig. 4). In the basic media, the hydroxyl ions attack the formed pyrrolone cation and convert it into the non-fluorescent, 2-hydroxy-pyrrolinone derivative. Conversely, in acidic media, the pyrrolone derivative is converted into lactone form [29], which has high structural similarity to fluorescamine. Owing to the non-planar structure of the 2-hydroxy-pyrrolinone derivative and lactone form (Fig. 4), their fluorescence activities are drastically reduced. Another possible cause for the variation in the fluorescence intensity upon changing the pH is the difference in nucleophilic activity which resulted from the change of the degree of protonation of the primary amino group [26].

After that, the procedure for general assay was carried out using varied volumes (0.2–1.2 mL) of borate buffer solution (pH 7.6) (Fig. 3). The highest results were achieved with 0.4–0.6 mL of the buffer solution and thus 0.5 mL of the borate buffer (pH 7.6) was recommended for the procedure in the general assay.

#### Influence of the concentration of fluorescamine

**Fig. 5:**
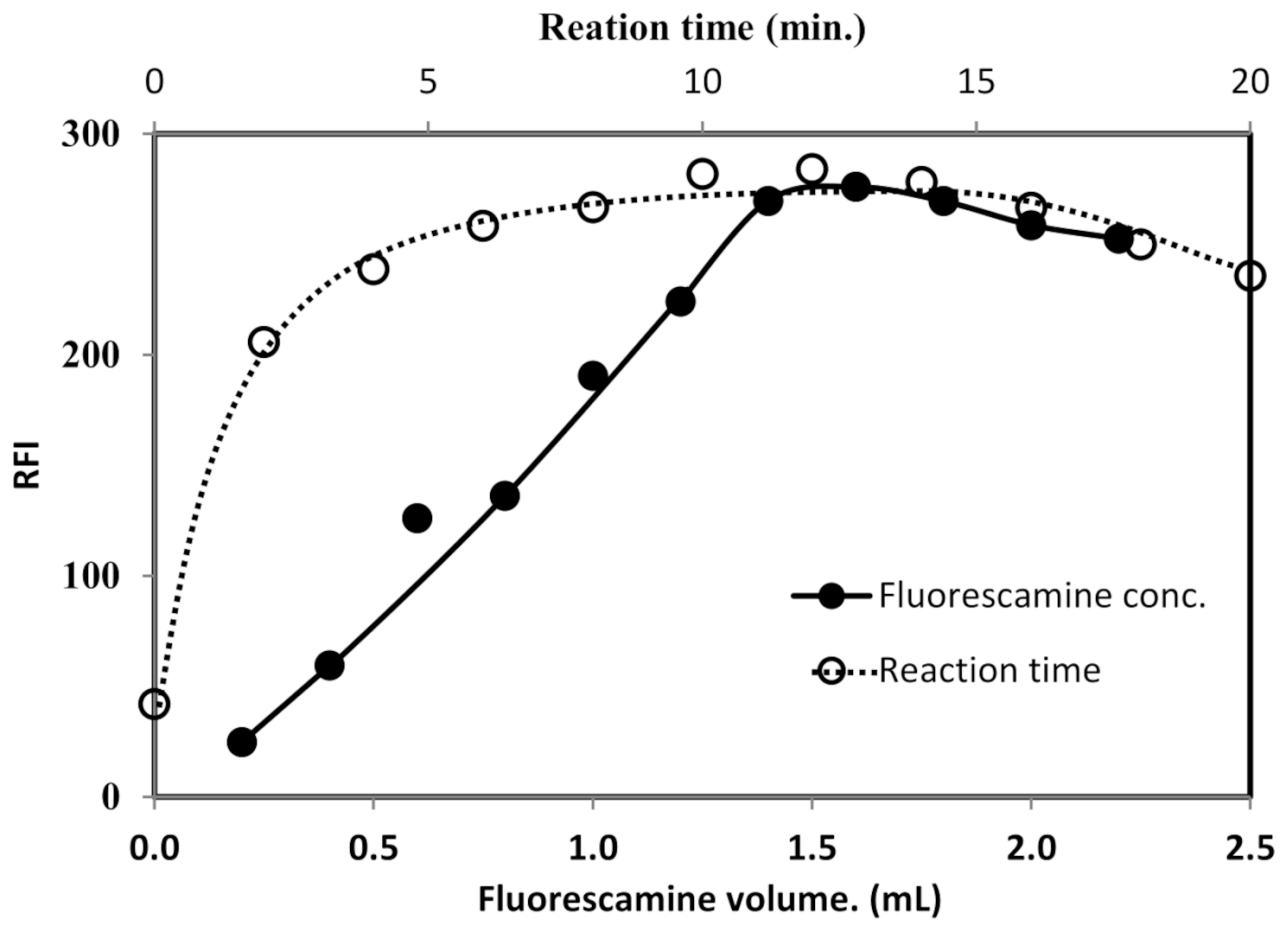
Effect of volume of fluorescamine reagent and reaction time on the RFI of the product of reaction between natamycin (2 µg/mL) and fluorescamine reagent

The reagent concentration influence on the fluorescence intensity was tested using varying volumes (0.2–2.2 mL) of fluorescamine solution that had a fixed concentration (200 µg/mL). The intensity of fluorescence was linearly increased when the volume of the reagent was increased. The intensity of fluorescence reached its maximum value when 1.4 mL was used. After that no significant changes in the fluorescence values were observed (Fig. 5). Thus, 1.6 ml of 200 µg/mL fluorescamine reagent was utilized in the analytical procedure.

#### Reaction time and product stability

The progress of the reaction was monitored by following the fluorescence intensity of the formed product at different time intervals. A rapid increase in the intensity of fluorescence was noticed upon mixing the reactants and a maximum value was attained within 6 min. This proved that the interaction between natamycin and fluorescamine was very rapid and reached to completion in a short time. Moreover, the fluorescence did not undergo any significant change for up to 20 min at ambient temperature (25 °C), (Fig. 5). Since the interaction was instantaneous at room temperature, therefore, there was no need for performing the reaction at an elevated temperature. Thus, the fluorescence intensity measurement was performed after keeping the reaction mixture at ambient temperature for 6 min.

#### Influence of dilution solvents

**Fig. 6:**
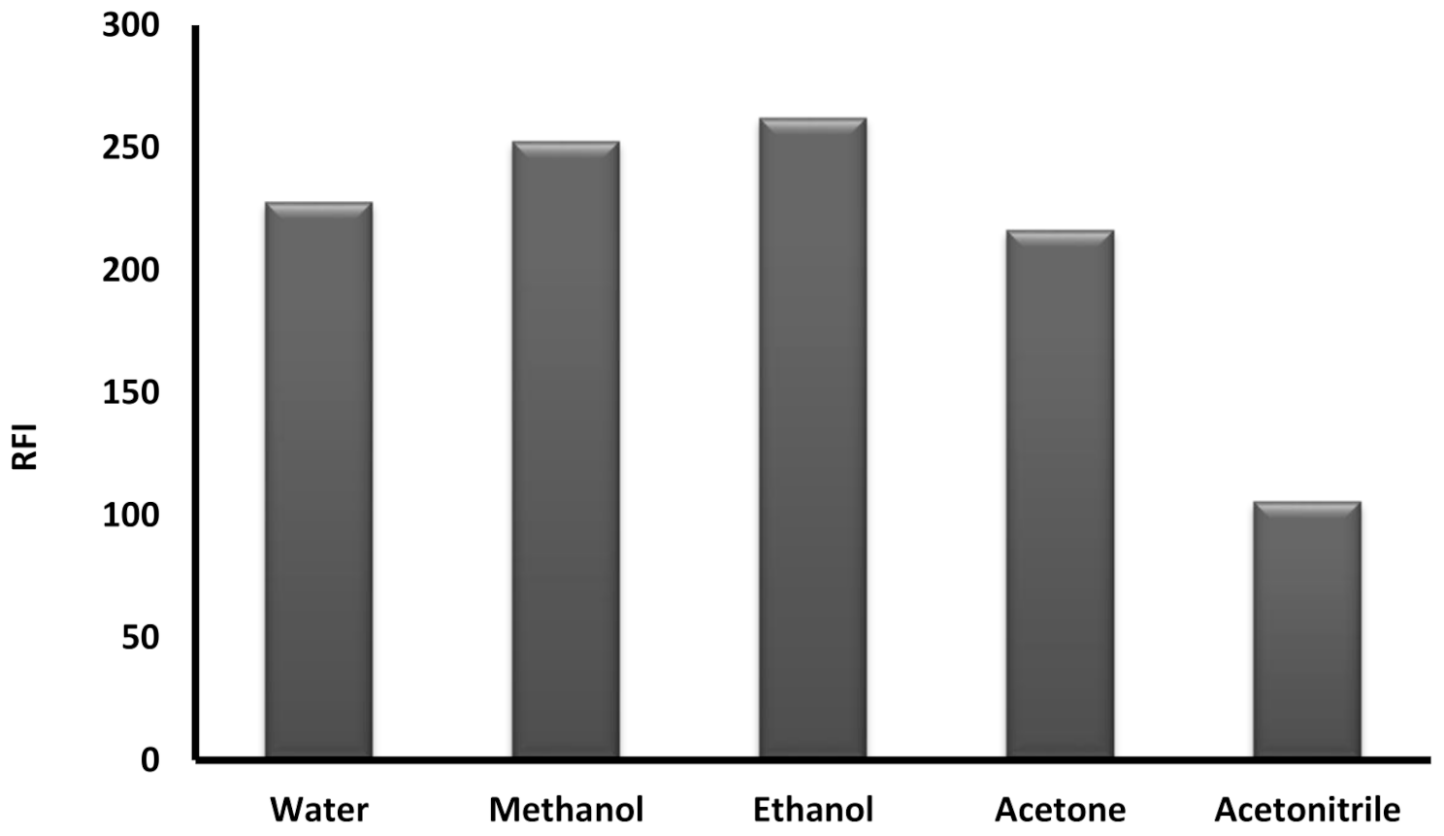
Effect of diluting solvent on the RFI of the product of reaction between natamycin (2 µg/mL) and fluorescamine reagent

To choose the best solvent for the reaction, various solvents were examined including acetone, acetonitrile ethanol, methanol, and water. Acetonitrile gave the lowest fluorescence intensity while the results of ethanol and methanol were the highest and were very close to each other, so ethanol was selected because of its lower toxicity and higher environmental safety than methanol (Fig. 6).

### Determination of the reaction stoichiometry

**Fig. 7:**
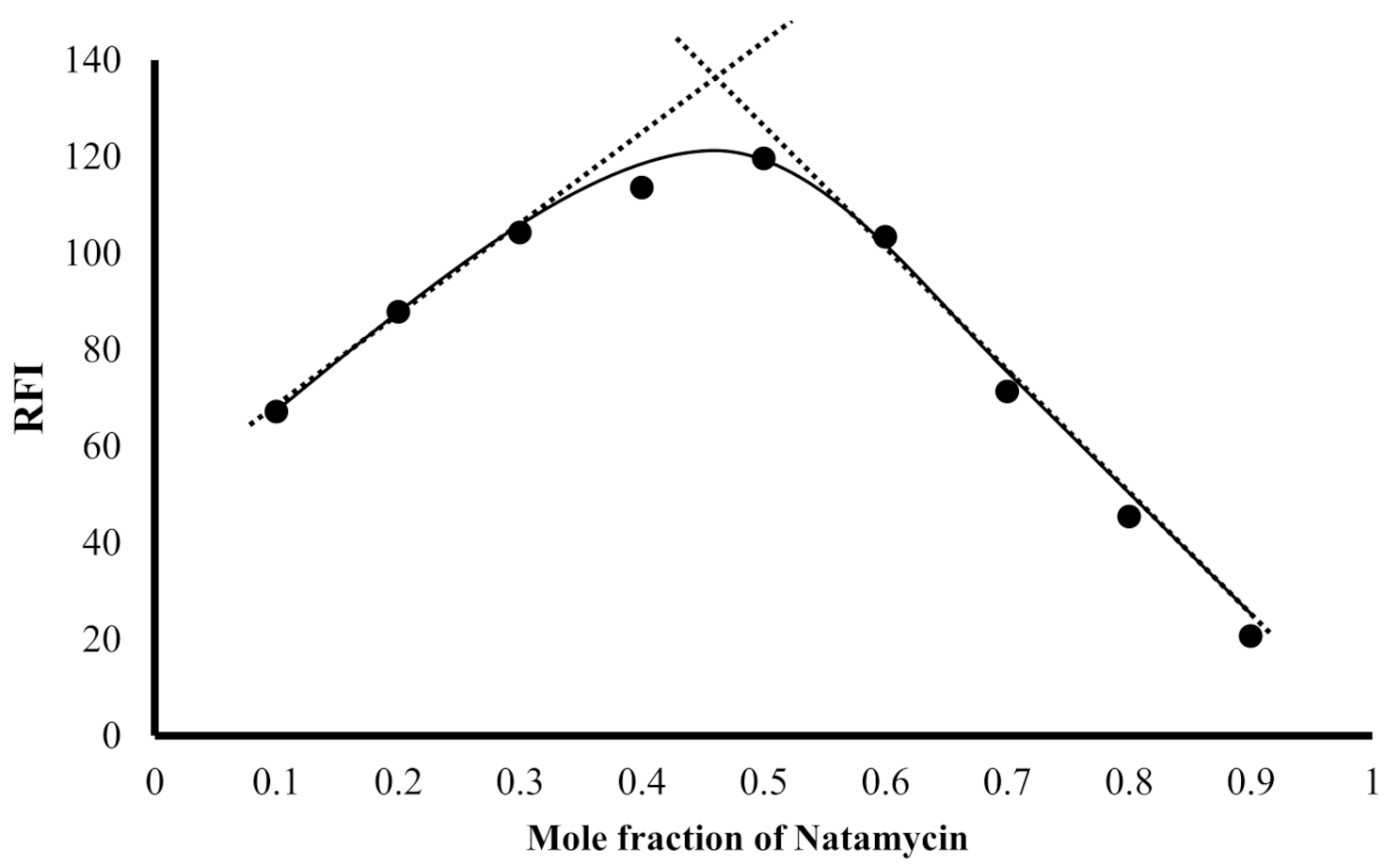
Job’s plot for determination of the stoichiometry of the reaction product using 1.2 × 10^− 4^ M concentration of both natamycin and fluorescamine

The stoichiometry of the reaction between natamycin and fluorescamine was determined using Job’s method. Solutions of equal molarity (1.2 × 10^− 4^ M) of both reactants were constituted and utilized in the general procedure. Job`s plot was built by relating the fluorescence intensity against mole fraction of the drug (Fig. 7). The highest fluorescence was obtained at 0.5 mol fraction of the drug which revealed molar ratio of 1:1 between natamycin and fluorescamine. The ratio is consistent with the presence of only one free amino group in the cited drug.

### Analytical method validation

Validation of the suggested procedure was done by applying the International Council for Harmonization (ICH) guidelines [30] in order to assess the linearity, range, accuracy, precision, specificity, selectivity, LOD and LOQ.

#### Linearity and range

**Fig. 8:**
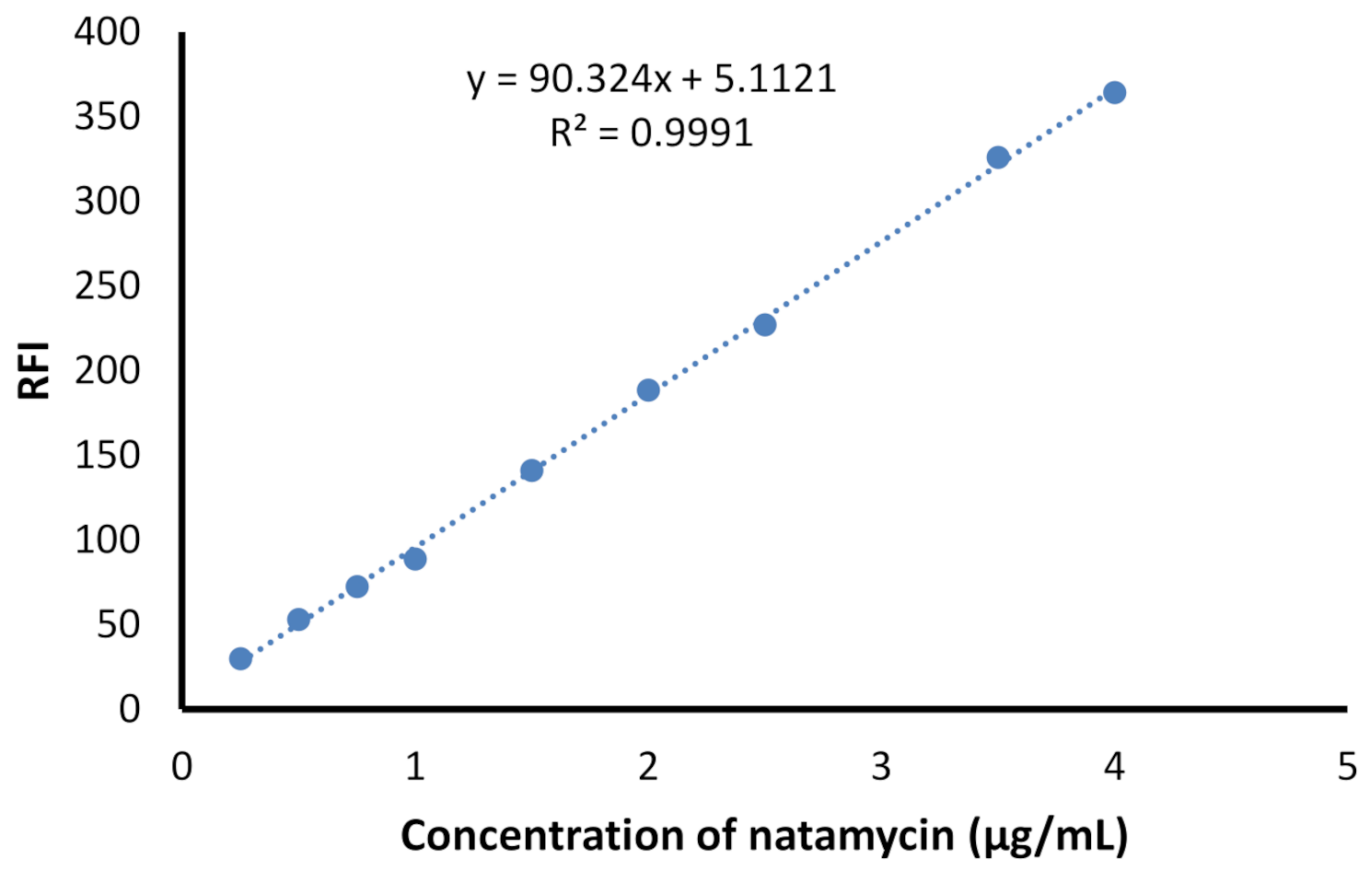
The calibration curve of the suggested spectrofluorimetric method for determination of natamycin via fluorescamine as a fluorogenic probe

**Fig. 9:**
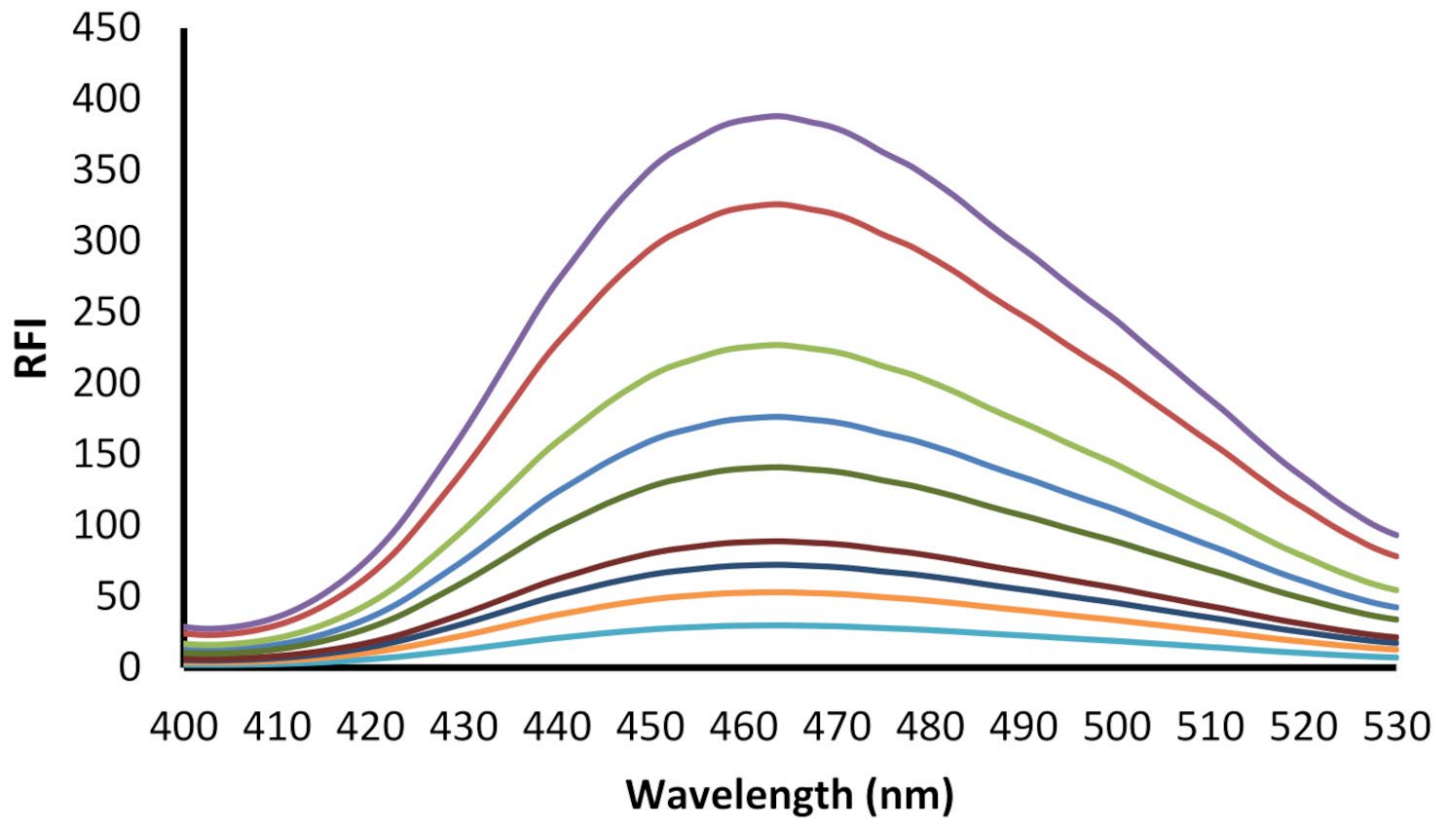
The fluorescence emission spectra of the reaction product between natamycin in the concentration range (0.25–4 µg/mL) and fluorescamine

After optimization of the experimental parameters, several working drug solutions having varied natamycin concentrations were subjected to analysis with the procedure of the general assay. The calibration plot was built by correlating the values of the fluorescence intensity with natamycin concentrations. The data were also processed statistically to find the linear regression equation using least square regression analysis. The slope, intercept, correlation coefficient are listed in Table 1. Figures 8 and 9 demonstrates the calibration curve and the fluorescence spectra corresponding to it, respectively. The linear range of the method was 0.25–4.0 µg/mL of natamycin with correlation coefficient of 0.9995 indicating the high linearity of the method. The low value of the standard deviation indicates the small scattering of the experimental points.

**Table 1:** The statistical parameters of the proposed method for analysis of natamycin

Parameter	Value
Linear range (µg/mL)	0.25–4.0
Slope	90.324
Standard deviation of slope (S_b_)	1.030
Intercept	5.112
Standard deviation of the intercept (S_a_)	2.241
Correlation Coefficient*	0.9995
Standard deviation of residuals (S_y, x_)	3.877
Limit of detection (LOD, µg/mL)	0.0819
Limit of quantitation (LOQ, µg/mL)	0.2481

#### Limits of detection and quantitation

To find the degree of sensitivity of the proposed spectrofluorimetric method, the limits of detection (LOD) and quantitation (LOQ) were computed. The slope (S) of the calibration curve and standard deviation (σ) of its intercept were utilized in these calculations by applying ICH equations; LOD = 3.3 σ/S and LOQ = 10 σ/S. The found LOD was 0.082 µg/mL and LOQ was 0.248 µg/mL, indicating the proper sensitivity of the suggested methodology. The achieved sensitivity was relatively better than the majority of spectrophotometric methods and also the spectrofluorimetric methods that involved the use of carbon dots or o-phthaldeheyde reagent [16, 18]. Moreover, the suggested approach was faster and simpler than the fluorimetric methods that involved Hantszch reaction as the latter reaction needed boiling for about half an hour [16, 17]. Also, the method involving the reaction of the drug with o-phthaldehyde required about 20 min that made the method more time consuming. Table 2 demonstrates a comparison between the suggested work and some of the reported fluorimetric methods. Furthermore, the ratiometric method [19] that utilized Eu@UiO-66(OH)2/(COOH)_2_ as a new fluorogenic probe needed very tedious and heavily time consuming probe synthesis that reached 48 h of heating and incubation in addition to multiple reagents preparations that opposed the simplicity of this method.

**Table 2:** Some reported spectrofluorometric methods compared with the suggested methodology

Reagent	Linear range (µg/mL)	LOD (µg/mL)	LOQ (µg/mL)	Heating Temperature	Time	Ref.
Acetyl acetone and formaldehyde (Hantzsch)	0.05–0.5 0.5-1.0	0.013 0.04	0.04 0.12	100^o^C 100^o^C	35 min 30 min	[16] [17]
o-phthaldehyde and N-acetylcysteine	0.5–10.0	0.16	0.48	Ambient	20 min	[16]
Nitrogen and sulfur carbon dots	0.5–10	0.10	0.45	Microwave for reagent preparation	4 min	[18]
Fluorescamine	0.25-4.0	0.082	0.248	Ambient	6 min	This work

#### Accuracy

To study the accuracy of the suggested methodology, three standard drug solutions with varied concentrations were mixed with a previously analyzed sample taken from the commercial eye drops solution. The obtained three different solutions were analysed using the procedure of the general assay. It was revealed from the obtained results that the present method has high degree of accuracy because the calculated % recovery values were close to 100% (Table 3). These results also indicated that the pharmaceutical excipients present in the eye drops formulation did not exhibit any interference in the obtained results giving a proof for the high selectivity of the current spectrofluorimetric approach.

**Table 3:** Standard addition method for the evaluation of the accuracy of the proposed method for the determination of natamycin in Hosaptam^®^ eye drops

Eye drops concentration (µg/mL)	Drug added (µg/mL)	Final taken concentration (µg/mL)	Found concentration (µg/mL)	% Recovery
0.5	0.25	0.75	0.739	98.56
0.5	0.5	1.00	0.989	98.93
0.5	0.75	1.25	1.201	96.14
Mean				97.88
SD				1.51
RSD				1.54

#### Precision

The precision of the current spectrofluorimetric approach was evaluated at two levels; intra- and Inter-day precision levels. Three standard solutions of different natamycin concentration levels (1.0, 1.5 and 2.0 µg/mL) were determined three times using the general procedure. The assay was performed within single day for intra-day precision and in three successive days for evaluating the precision at inter-day level. The results showed good % recoveries and low % relative standard deviation (RSD). The RSD was taken as a measure for the precision level. As illustrated in Table 4, the values of RSD were not higher than 2% which confirmed the excellent precision level of the proposed methodology.

**Table 4:** Evaluation of the intra-day and inter-day precision of the proposed method for the determination of natamycin at three concentration levels

Concentration level (µg/mL)	% Recovery* ± RSD	
	Intra-day precision	Inter-day precision
1.0	98.79 ± 1.99	100.36 ± 1.52
1.5	98.33 ± 0.93	99.76 ± 1.30
2	101.07 ± 1.19	102.64 ± 1.35

^a^ The value is a mean of three determinations

#### Robustness

The robustness of the proposed method was confirmed by making little changes in some of the reaction conditions (volume of buffer, pH, fluorescamine volume and time of the reaction). In each case, the %recovery value was estimated as well as the RSD. It is obvious from the data in Table 5 that the analytical response of the method was not significantly altered by such alterations, as the recovery percentage was good, and the RSD value was low. Thus, the suggested method is considered robust.

**Table 5:** Assessment of the robustness of the proposed method for analysis of natamycin (3 µg/mL)

Optimization Factor	Value	% Recovery ^a^	Mean ± SD	% RSD
Borate buffer pH	7.5	97.88	98.89 *±* 1.04	1.08
	7.6	96.98		
	7.7	95.80		
Borate buffer volume (mL)	0.4	98.52	97.38 ± 1.23	1.26
	0.5	97.52		
	0.6	96.08		
Reaction time (minutes)	5	99.63	101.77 ± 1.87	1.83
	6	102.65		
	7	103.03		
Fluorescamine volume (mL)	1.4	97.67	98.43 ± 1.35	1.38
	1.6	99.99		
	1.8	97.61		

^a^ this value is a mean of three determinations

### Method applications

#### Analysis of eye drops formulation

The proposed method was employed to estimate the natamycin content in Hosaptam^®^ eye drops. The prepared solution of the eye drops (of concentration 50 µg/mL) was diluted with the appropriate volume of methanol to obtain a solution of 10 µg/mL of natamycin. One millilitre portion of the resulting solution was analyzed by the general procedure (giving final concentration 1.0 µg/mL). Another diluted sample solution of 25 µg/mL concentration was also analyzed by a reported HPLC method with ultraviolet detection [6]. The found mean %recovery for the suggested method was 102.24%, and the standard deviation value was 1.79. Comparison of the results obtained from the analysis of Hosaptam^®^ eye drops for both proposed and reported methods was carried out using Student t- and F- tests. The calculated values of the two parameters were found to be lower than the tabulated values. Hence, there is no considerable alteration between both methods regarding accuracy and precision (Table 6).

**Table 6:** Analysis of natamycin in Hosaptam^®^ eye drops using the proposed and the reported method

Parameters	Proposed method (Taken amount 1 µg/mL)		Reported method [6] (Taken amount 25 µg/mL)	
	Amount Found	% Recovery	Amount Found	% recovery
% Recovery	1.035	103.55	25.072	100.29
	1.019	101.95	25.217	100.87
	0.993	99.29	25.200	100.8
	1.027	102.66	24.924	99.69
	1.037	103.73	24.169	96.68
Mean recovery		102.24		99.67
Standard deviation		1.79		1.74
t-Student value ^a^		2.301		
F variance value ^a^		1.070		

^a^ Tabulated values at 95% confidence limit are t = 2.306, F = 6.338

#### Analysis of aqueous humour

Artificial aqueous humour was prepared and spiked with natamycin in order to simulate the real human aqueous humour. Due to the high sensitivity of the suggested spectrofluorimetric method, it was tried for the determination of natamycin in spiked artificial aqueous humour. The aqueous humor was artificially prepared according to the previously published method [31]. A portion of the sample was spiked with the cited drug in three concentration levels. Precipitation of the albumin with methanol was necessary to avoid its possible interferences. Thus, an equal volume of methanol was added, and the solution was centrifuged for 15 min at 3000 rpm. Aliquots of the resulting supernatants were analyzed with the proposed spectrofluorimetric method. The concentrations of the studied drug in the spiked samples were calculated from the linear regression equation. Good % recoveries were obtained with low values of standard deviations (Table 7). These results proved the method’s accuracy, precision and selectivity. The high %recovery values also indicated that the components of the aqueous humor did not have any notable influence on the results of the developed method. Therefore, the method can be used for the determination of natamycin in human aqueous humour.

**Table 7:** Application of the proposed method spiked artificial aqueous humour

Concentration level (µg/mL)	% Recovery ^a^ ± RSD
1.0	99.59 ± 0.58
2.0	104.63 ± 1.29
3.0	99.14 ± 1.34

^a^ The value is a mean of three determinations

### Assessment of the method greenness

Analysts have a great deal of responsibility to mutually protect the environment and people from harmful chemical substances and waste produced by their activities [32]. The design and upgrade of green chemistry should be done in a regular manner. State-of-the-art consideration, such as ecology scale scores [33] and Analytical Greenness Calculator (AGREE) [34], were used to evaluate the “environmental value” of the analytical method. In the present study eco-scale was utilized to find out how green the proposed methodology is. The results of the eco-scale evaluation are represented in numbers indicating issued penalty points subtracted from 100. The points express the risks encountered during carrying out the intended procedure. The higher the score is (expressed by a large number), the greener the procedure. The suggested methodology did not involve any extraction, or heating steps. In addition, the energy consumption of the procedure was lower than 0.1 kW/h for a single sample. Consequently, the proposed method has a score in eco-scale of 90 (Table 8) which reflects clearly the environmental sustainability of the utilized technique.

**Table 8:** Penalty points calculation for the greenness evaluation of the present method

Item	Parameter	Word sign	PP score
Technique	Spectrofluorimetry	LSH	0
Reagent(s)	Fluorescamine	MSH ^*^	1
Amount of reagent	> 10 mL		1
Solvent(s)	Acetone (for flurescamine) Ethanol (for dilution)	MSH LSH	4 1
Heating	No heating		0
Temperature	Ambient temperature		0
pH	7.6		0
Cooling	No cooling		0
Energy (kWh per sample)	> 1.0		0
Waste	1–10 mL		3
Occupational hazards			0
(TPPs)			10
Eco-scale total score	= 100 – TPP		90

^*^ MSH is an abbreviation for the More severe hazard, LSH for the Less severe hazard, and TPPs for the Total penalty points

**Fig. 10:**
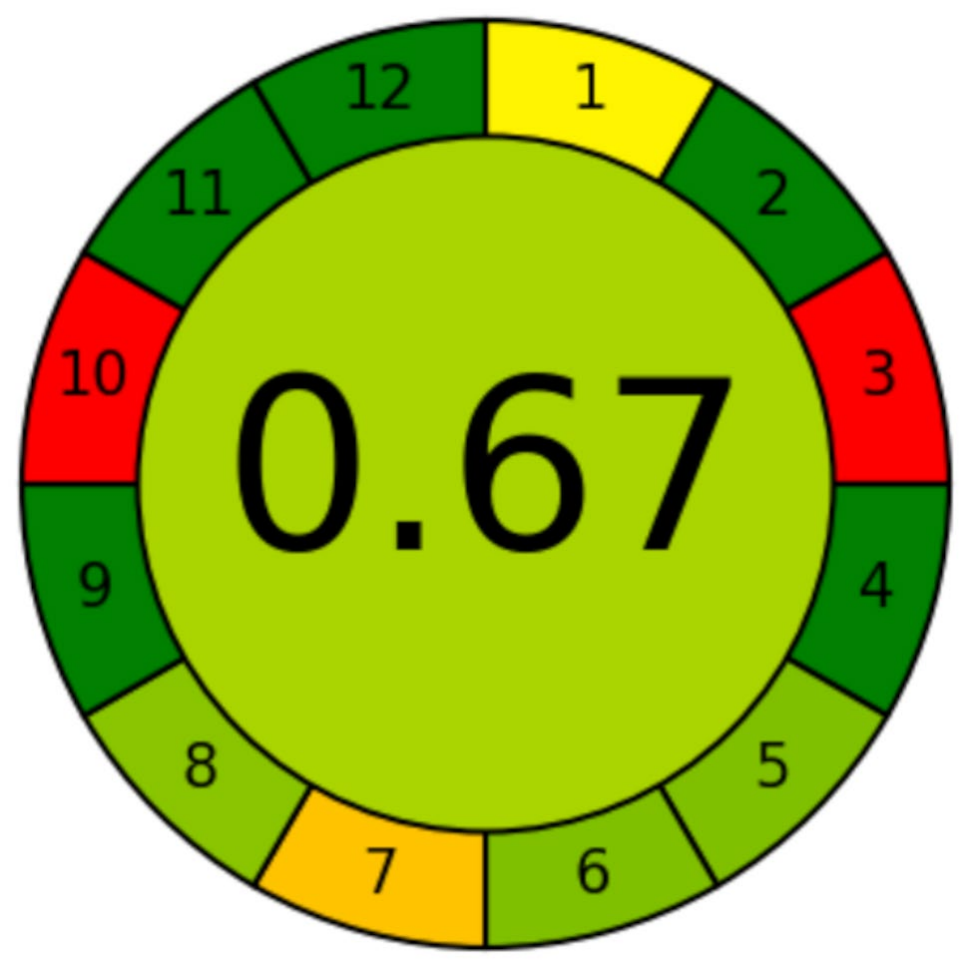
Greenness evaluation of the suggested spectrofluorimetric methods using AGREE tool

The AGREE software is an easy-to-use application that uses the green analytical chemistry twelve-significance principles as its input criteria. On a conventional scale from 0 to 1, each of these twelve inputs is assigned a score that appears on an easy-to-understand red-yellow-green color scale. Furthermore, the procedure takes into account the significance of each input criterion, which is represented by the width of the segment that corresponds to it. The output is displayed as a graph that resembles a clock, with the overall score and color representation at its center. A dark green tint indicates a perfect analysis, which receives a score of one. The current spectrofluorimetric approach achieved an exceptional green analysis with a score of 0.67, as shown by the AGREE pictogram (Fig. 10).

## Conclusion

The present work utilized a feasible reaction based on derivatization with fluorescamine reagent for the rapid and simple spectrofluorimetric determination of natamycin. The procedure did not involve any drastic conditions or multistep reaction. Sample preparation is straightforward and did not include any tedious or lengthy procedure. The method was employed with great success for the assay of natamycin in commercial eye drops and artificial aqueous humour. Therefore, the suggested methodology could be a valuable alternative for natamycin assay in quality control because of its high sensitivity, simple procedure and using relatively inexpensive instruments.

## Data Availability

All data underlying the results are available as part of the article and no additional source data are required.

## Abbreviations

LOD: Limit of detection
LOQ: Limit of quantitation
HPLC: High performance liquid chromatography
UPLC: Ultraperformance liquid chromatography
RSD: Relative standard deviation
ICH: International Council for Harmonization
S: Slope
AGREE: Analytical Greenness Calculator
